# The Irish COPD paradox and the promise of virtual care

**DOI:** 10.3389/fdgth.2026.1838140

**Published:** 2026-05-29

**Authors:** Emma Lynn Burke, Clare Connolly, Niki Byrne, Karolina Glomba, Ian McCabe, David Tiernan, Sheila Gleeson, Hemendra Worlikar, Derek OKeeffe, Sinead Walsh

**Affiliations:** 1 Respiratory Department, Galway University Hospital, Galway, Ireland; 2 Respiratory Integrated Care, Galway City Hub, HSE West Northwest, Galway, Ireland; 3 HIVE Lab, University of Galway, Galway, Ireland

**Keywords:** COPD—chronic obstructive pulmonary disease, digital health equity, health system integration, hospital at home, inverse care law, mixed methods feasibility study, RPM (Remote patient monitoring), virtual care pathway

## Abstract

**Introduction:**

Ireland has the highest COPD hospitalisation rate in the OECD (315 per 100,000 vs. an average of 190), yet possesses the infrastructure and reform ambition—through Sláintecare—to deliver care differently. Virtual Care Pathways underpinned by Remote Patient Monitoring offer one route out of this costly, hospital-centric cycle, but the question is not simply whether they work. It is how, for whom, and at what cost to equity.

**Methods:**

We conducted a 20-month prospective mixed-methods feasibility study at Galway University Hospital, enrolling 85 adults with high-risk COPD (GOLD Group B/E) into a nurse-led, protocol-driven Virtual Care Pathway using tablet-based remote monitoring with 5G connectivity. Clinical outcomes were evaluated against historical baselines; patient experience was explored through focus groups and serial surveys, with findings interpreted through a critical realist lens and the NASSS framework.

**Results:**

Of 152 exacerbation episodes managed on the platform, 148 (97.3%) were completed without hospital admission. Mean length of stay was 5.15 days—a 51.5% reduction against the regional baseline of 11.8 days (*p* < 0.001)—translating to an estimated €949,000 in gross hospital cost avoidance. Borg dyspnoea and CAT scores improved significantly beyond minimal clinically important differences. But the qualitative data complicates this picture.

**Discussion:**

Patients embraced the platform largely because hospital terrified them, not because the technology delighted them. The “digital safety net” generated its own anxieties around device failure and clinical abandonment, and families—particularly daughters and grandchildren—absorbed a hidden burden of technical troubleshooting that the model depends on but does not account for. Most critically, every participant owned a smartphone. In a country where 37% of over-65s are digitally excluded, and where COPD prevalence is itself socially patterned, the absence of digitally excluded patients from our sample is not a limitation to footnote—it is the finding. Without deliberate design of hybrid digital-analogue pathways, Ireland risks cementing an “Inverse Digital Care Law” in which the most effective care reaches those who need it least.

## 1 Introduction: the Irish COPD paradox and the promise of virtual care

Chronic Obstructive Pulmonary Disease (COPD) remains a challenging and escalating global health challenge, ranking as the third leading cause of death worldwide and a major driver of morbidity and healthcare expenditure (1, 2). Its progressive and unpredictable nature, characterised by persistent respiratory symptoms and irreversible airflow limitation, necessitates long-term, complex management that ideally fits across community and acute care settings. This management is complicated by the disease's trajectory, characterised by acute exacerbations (AECOPD)—events of acute worsening of respiratory symptoms that lead to significant morbidity, mortality, and healthcare utilisation. Within this global context, Ireland presents a noteable and paradoxical case study among high-income nations. Despite possessing a robust healthcare infrastructure and a relatively small, well-defined population, Ireland endures one of the heaviest and most costly COPD burdens in the Organisation for Economic Co-operation and Development (OECD). Recent data reveals the country has the highest hospitalisation rate for COPD across OECD members, at 315 admissions per 100,000 population, a figure starkly contrasted against an OECD average of 190 (3). This disparity is not merely statistical but represents profound human and systemic cost. It is compounded by significant and persistent regional inequities, with the length of stay (LOS) for COPD admissions varying dramatically from an average of 6.5 days in some hospital groups to over 12 days in others, pointing to fragmented, inconsistent, and often inefficient care pathways nationwide (4).

This persistently high hospitalisation rate is symptomatic of a historically embedded, hospital-centric model of care, a system ill-suited for the proactive, longitudinal management of chronic conditions. The Irish health system, like many globally, has traditionally been configured around episodic, acute interventions—a structure that fails to meet the continuous, preventative, and personalised needs of individuals with COPD. Consequently, the management of AECOPD—events that are frequently preventable or manageable in community settings with early intervention—consumes a disproportionate and unsustainable approximately 70% of the national respiratory care budget (5). This reality is a textbook manifestation of the “inverse care law”, first proposedby the pioneering general practitioner Julian Hart in 1971, which holds that the availability of good medical care tends to vary inversely with the need for it in the population served (6). In the Irish COPD context, this law is clearly operational. Those with the greatest socio-economic and clinical need for structured, accessible COPD management—often older adults, those in rural areas with limited primary care access, or individuals from lower socio-economic groups—frequently experience the most disjointed access to proactive care. This results in late, crisis-driven presentations to overcrowded emergency departments, leading to costly hospital stays, thereby demonstrating a vicious cycle of declining health and system strain (7).

Ireland's ambitious, decade-long Sláintecare reform programme aims to dismantle this paradox by fundamentally re-orienting the health system towards integrated, population-based, and equitable care. Its core vision, is to provide the “right care, in the right place, at the right time” by decisively shifting care delivery from institutional hospitals to the community and enhancing preventative and primary care services (8). Virtual Care Pathways (VCPs), powerfully underpinned by Remote Patient Monitoring (RPM) technologies, emerge as a vital and potentially transformative enabler of this vision. By allowing patients to securely transmit physiological data (e.g., respiratory rate, temperature, oxygen saturation, heart rate and blood pressure) and symptom scores [e.g., COPD Assessment Test (CAT) and breathlessness scoring tools such as mMRC] from their homes to a coordinating clinical team, VCPs facilitate the early detection of physiological deterioration pre-empting an exacerbation. This enables timely, targeted interventions—such as medication adjustment, a specialist nurse visit, multi-disciplinary meeting discussion or a GP consultation—potentially averting the need for a hospital admission entirely. International evidence robustly supports this potential; high-quality systematic reviews and meta-analyses indicate that well-structured telemedicine and RPM interventions for COPD can lead to statistically significant reductions in hospital admissions, emergency department visits, bed-day use, and improvements in health-related quality of life (HRQoL) (9–11). Early Irish pilot studies, though typically small-scale and of limited duration, also show promise, consistently reporting high patient satisfaction and preliminary signals of reduced healthcare utilisation (12).

However, the translation of these promising, contained pilots into scalable, equitable, and sustainable national pathways is complicated by gaps that the Irish system must confront. First, there is the significant challenge of transferability and contextual fit. Systems like Kaiser Permanente (US) and the UK's NHS, which are built on integrated care and strong primary care, may not align with Ireland's fragmented landscape of mixed funding, historically low levels of primary care investment and staffing, and uneven regional infrastructure (13). Second, and perhaps most importantly, there is a growing risk of worsening the existing health disparities. The digital transformation of healthcare, while offering immense potential, can inadvertently create or widen a “digital divide”. In this scenario, innovations preferentially benefit those with higher digital literacy, better broadband connectivity, and higher health literacy, thereby intensifying Hart's inverse care law into what scholars term an “inverse digital care law” (7). This risk is particularly acute and pertinent in Ireland, where significant digital exclusion persists, especially among older populations, those with lower educational attainment, and people in socio-economically disadvantaged or rural areas (14). Third, the long-term economic viability and business case for national scaling remains uncertain. While RPM may avert costly acute hospital admissions, its sustainable scaling requires substantial upfront and recurrent investment in technology, comprehensive workforce redesign, and the development of new funding models that actively reward keeping people well at home, rather than the current activity-based funding that incentivises hospital treatment (15, 16). The financial benefits (cost avoidance) often accrue to the hospital budget, while the costs of running the VCP may fall on community or new innovation budgets, creating a resistance for adoption.

Therefore, this pilot study consciously moves beyond a simple evaluation of clinical outcomes. It adopts a critical realist research lens to investigate not merely if a VCP works in a controlled setting, but how, for whom, and under what conditions it functions, fails, or adapts within Ireland's complex, multi-layered health ecosystem. Critical realism, a philosophy of science developed by Roy Bhaskar, distinguishes between three domains: the empirical (what we directly observe and experience), the actual (all events that occur, whether observed or not), and the real (the underlying structures, powers, and mechanisms that generate events) (17). Applied to health services research, this perspective seeks to uncover the deep-seated social, technological, organisational, economic, and clinical mechanisms that enable or constrain the implementation and impact of innovations like VCPs (18). This study specifically focuses on the core tension between the technological promise of integrated, patient-centred care and the reality of systemic fragmentation. It also critically examines a key conflict. On one hand, innovation promises to give patients more control; on the other, it risks leaving behind those who cannot access or use digital technology, thereby deepening existing divides. By looking at what drives these outcomes, the study aims to produce not just findings, but transferable knowledge and theories with defined scope for policymakers, clinicians, and service designers seeking to navigate Ireland's digital health transformation in a responsible, equitable, and effective manner.

## 2 Methods

### 2.1 Study design and theoretical framework

This was a prospective, mixed-methods feasibility study conducted from April 2024 to December 2025 (20 months).This study was based on the view that the success of the VCP depends on more than just technology or patient traits. Instead, it considers the complex real-world contexts in which the program operates. This study recognised that the outcomes of the VCP resulted from a complex mix of factors: technology, individual choices, social dynamics, organisational structures, and policy. To analyse this complexity, we used the NASSS framework. This toolkit is designed to understand why complex health technologies succeed or fail (18). The NASSS framework, is a practical utility that prompts investigators to systematically consider complexity and uncertainty across seven interdependent domains (Table 1).

**Table 1:** NASSS framework domains applied to the study.

Domain	Description & application to the COPD RPM study
1. The Condition	COPD as a chronic, variable, and socially patterned illness.
2. The Technology	The RPM kits (hardware) and software platform, including their capabilities and limitations.
3. The Value Proposition	Perceived benefits, costs, and risks for patients, clinical staff, and the health system.
4. The Adopter System	**Patients**: Capacities, motivations, and life contexts. **Staff**: Roles, skills, and attitudes towards RPM.
5. The Organisation(s)	The implementing hospital: its internal processes, workflows, and readiness for change.
6. Wider Context	Policy (Sláintecare), funding flows, regulations, and national digital infrastructure.
7. Embedding & Adaptation	How the technology and its use evolved over the study timeline.

Our mixed-methods design allowed us to generate data for each of these domains, to explore not just correlation but causation within a complex system.

### 2.2 Population, participants, and the context of selection bias

Participants (*n* = 85), total number of episodes 159, were recruited from the respiratory outpatient department and acute hospital of Galway University Hospital (GUH), a large tertiary referral centre and a key component of the HSE West Northwest in Ireland. Recruitment was strategically targeted at the catchment area of Community Healthcare Networks 4, 5, 6 (CHN). This ensured logistical feasibility and, guaranteed that all enrolled patients remained under the established consultant governance and care team. Moving outside this area would have introduced different consultants and staff, fracturing the continuity of care essential for the study.

Inclusion Criteria were designed to identify a cohort most likely to benefit from and safely engage with the VCP:

1. Age ≥18 years with a spirometry-confirmed diagnosis of COPD (Post-bronchodilator FEV1/FVC <0.70) (19).
2. High risk of exacerbation, defined as GOLD classification Group B or E (symptomatic patients), with a history of ≥2 moderate or severe exacerbations in the previous 12 months, indicating a high likelihood of future events (20).
3. Geographical residence within the defined CHN, ensuring the feasibility of emergency response or community health intervention if urgently required.
4. Demonstrated capacity and willingness to use the RPM technology after a standardised, hands-on training session conducted by the VCP team members.
5. Physiological stability at enrolment (resting SpO₂ > 88% on room air, heart rate <100 bpm, and not in acute respiratory failure) to ensure safe initiation of home monitoring.

Exclusion Criteria were applied for safety and protocol integrity: significant cognitive impairment (MMSE <24: Scores of 24 or above generally suggest no significant cognitive impairment) effecting informed consent or safe use of technology; a primary diagnosis of another dominant respiratory disease (e.g., severe asthma, cystic fibrosis); active palliative care needs where the treatment goal shifted to end-of-life care; or lack of a suitable home environment (e.g., no reliable electricity).

The recruitment criteria, particularly criteria 4 and 5, introduced an inherent selection bias—a key methodological limitation and ethical consideration. While necessary for a feasibility pilot, it created a cohort that was inherently more digitally capable and clinically stable at point of entry than the broader COPD population. We recorded the number of patients screened and excluded for these reasons, as this data itself is a vital finding regarding the “real-world” applicability of such digital solutions.

Written informed consent was obtained from all participants. Ethical approval was granted by the HSE University Hospital Galway Ethics Board.

### 2.3 The intervention: a detailed overview of the COPD virtual care pathway

The intervention was a comprehensive, protocol-driven, and respiratory specialist nurse-led COPD Virtual Care Pathway, conceptualised as a “hospital-at-home” service for exacerbations (21). The pathway was co-designed with initial patient and clinician input prior to launch (22).

Technology Provision: Upon enrolment, patients were provided with a bespoke RPM kit housed in a dedicated carry case. The kit comprised (Table 2):

**Table 2:** Remote patient monitoring (RPM) kit components.

Device	Model & specifications	Primary function/data collected	Connectivity & integration
Tablet Computer	Samsung Galaxy Tab A8 (5G-enabled)	Served as the data hub and user interface; hosted the MyPatientSpace (MPS) platform.	5G Cellular; Integrated all peripheral device data via the MPS app.
Blood Pressure Monitor	Omron M7 Intelli IT	Automated measurement of systolic and diastolic blood pressure.	Bluetooth to tablet.
Pulse Oximeter	Masimo MightySat® (medical-grade)	Measurement of peripheral oxygen saturation (SpO₂) and respiratory rate.	Bluetooth to tablet.
Thermometer	iHealth PT3	Measurement of body temperature.	Bluetooth to tablet.

A dedicated helpdesk number and a simplified paper-based backup system were also provided.

**Figure 1:**
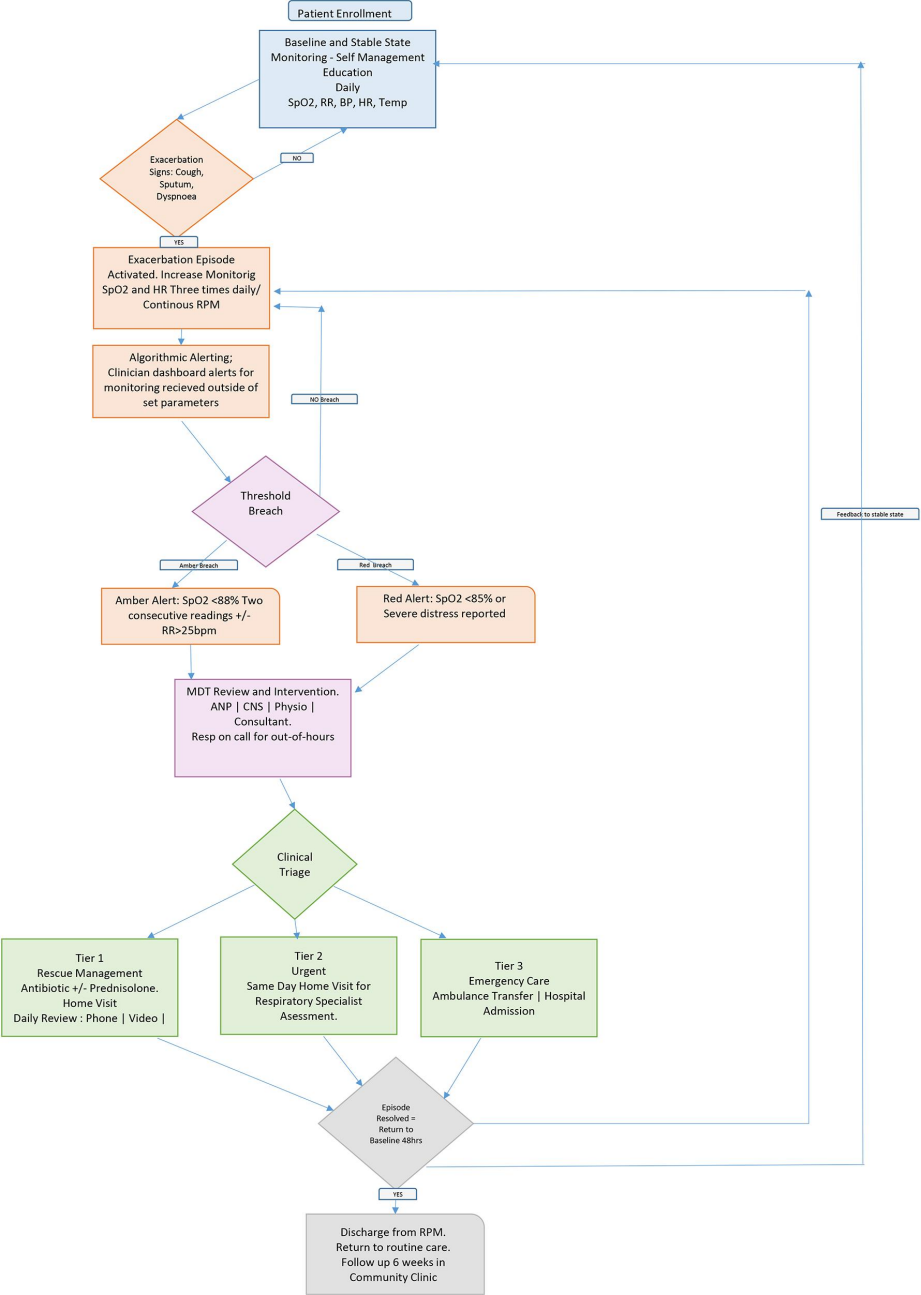
Remote patient monitoring (RPM) workflow for COPD exacerbation management. The process illustrates the transition from stable state baseline monitoring to intensified surveillance during suspected exacerbations. Automated algorithms detect threshold breaches (amber/red) to trigger MDT discussion, leading to tiered interventions (home management, urgent assessment, or emergency care). Episode resolution requires 24 h of stable data prior to discharge to routine care with 6-week follow-up.

The protocol (operationalised in Figure 1) was structured to mirror the clinical reasoning of a hospital admission but in a home setting. Patients enrolled in the Virtual Care Pathway were required to perform and transmit a set of vital signs and symptom scores twice daily (between 08:00–10:00 and 18:00–20:00) throughout the acute exacerbation episode. The mandatory daily dataset included; peripheral oxygen saturation (SpO₂), respiratory rate, heart rate, blood pressure, temperature, Borg dyspnoea score, and COPD Assessment Test (CAT) score. Patients were also asked to record sputum colour and volume if applicable.

Measurements were transmitted automatically via Bluetooth to the MyPatientSpace tablet, which relayed data to a secure clinician dashboard. Patient-initiated measurements were permitted at any time if the patient felt they were deteriorating, but the protocol required scheduled readings as the minimum standard: a full set once daily and SpO₂ three times per day.

The clinical dashboard was reviewed thrice daily (09:00, 12:00 and 16:00) on weekdays by the Advanced Nurse Practitioner (ANP). The Pathfinder Service—a regional acute medical remote triage service run by the National Ambulance Service—provided weekend and out-of-hours cover for acute patients. This included weekdays (16:00 to 00:00), and weekends (12:00 to 00:00). The service was activated once patients were identified by the Virtual Care Pathway team on a Friday evening (16:00).

Patients triggering an amber alert received a telephone review by the ANP within 2 h, with advice on medication adjustment (e.g., increased bronchodilator use, commencement of oral corticosteroids if not already prescribed). Patients triggering a red alert were contacted immediately; if clinical deterioration was confirmed, the patient was advised to attend the Emergency Department or trigger Pathfinder service.

Each scheduled monitoring session required approximately 10–15 min of patient time (based on post-study survey data). Patients who encountered technical difficulties could use a paper-based backup diary (daily readings transcribed and transmitted via telephone to the ANP) or request a home visit from the VCP team. These alternatives were used in 12% of episodes (*n* = 18) on occasion.

### 2.4 Data collection and analysis: a mixed-methods approach

A mixed-methods design was employed to provide a comprehensive, multi-faceted evaluation, aligning with the realist journey of understanding different layers of reality (empirical, actual, and real). Data collection instruments were mapped to the NASSS domains.

#### 2.4.1 Quantitative data collection & analysis

The primary outcomes focused on the pathway's impact on hospital utilisation. Hospital admissions avoided were defined as the count of exacerbation episodes managed entirely on the virtual care platform without requiring a physical hospital admission, serving as a direct measure of the programme's ability to “shift left” care delivery. Length of stay was defined as the duration, in days, from the virtual care platform episode initiation to formal discharge from enhanced monitoring back to usual care. These data were analysed using descriptive statistics, with mean values and ranges calculated to summarise observed trends and compared against a pre-intervention baseline derived from Hospital In-Patient Enquiry data for similar-severity COPD admissions from the same catchment area between 2019 and 2022 (4). Severity matching was based on the presence of two or more relevant comorbidities (e.g., cardiac disease, diabetes, renal impairment) recorded in HIPE, which classifies such admissions as “major severity”. This approach provides a pragmatic, real-world benchmark rather than a direct case–control comparison. Secondary outcomes comprised patient-reported outcome measures collected at episode initiation and again at discharge. These included the Borg Dyspnoea Scale to assess perceived breathlessness (23), the COPD Assessment Test to evaluate symptom burden (24), and the EQ-5D-5L to capture generic health-related quality of life and generate utility scores for potential economic evaluation. Patient-reported outcome measures were examined by comparing mean scores at episode initiation and discharge. A conservative cost-avoidance analysis was also undertaken, wherein avoided inpatient bed days were multiplied by the National Costing Programme's Diagnosis-Related Group rate for a COPD admission without major complications (DRG E65A: €6,411), acknowledging that this gross avoidance figure does not offset virtual care platform operational costs such as staffing, technology, and overheads (14, 16). To provide a balanced assessment, we estimated the costs incurred by the Virtual Care Pathway. Based on detailed service costing, the annual non-pay (technology) costs comprised platform licence for 250 patients (€20,500), device management and connectivity (€24,000), and technical support (€8,900), totalling €53,400 per annum. Staffing costs included 1.0 WTE Advanced Nurse Practitioner (€93,660) and 0.5 WTE administrative support (€26,445), giving a total annual pay cost of €120,105. The combined annual service cost was therefore €173,505. Over the 20-month pilot (pro-rated), this equated to approximately €289,175 total service cost.

On a per-patient basis, with 159 episodes of care over 20 months (approximately 80 patients per year), the average non-pay (technology) cost was €672 per patient per year, and the total cost (including staff) was €2,182 per patient per year or €1,068 per treated episode (Table 3). These figures represent the gross costs of service delivery and do not include hospital overheads or primary care costs. A full incremental cost-effectiveness analysis, including sensitivity analyses around staff time attribution and volume scaling, is planned but beyond the scope of this feasibility pilot. For the purpose of this analysis, we assumed that exacerbations meeting any of the following criteria during RPM monitoring would have required hospital admission under standard care: failure to improve within 48 h of self-initiated rescue medication, a sustained fall in SpO₂ > 4% from baseline, purulent sputum with positive culture, or clinical deterioration prompting multidisciplinary team escalation. This assumption is deliberately conservative for cost-avoidance estimation but does not imply that every RPM-managed exacerbation would have resulted in admission. A proportion may have been managed in primary care or resolved without escalation.

**Table 3:** Cost breakdown and return on investment.

Metric	Technology only	Including full staff attribution
Total investment over 2 years	€106,800	€347,010
Cost per patient per year	€1,068	€1,735
Cost per episode	€672	€2,182
Hospital cost avoidance	€984,139	€984,139
Net cost avoidance	€877,339	€637,129
Return on investment (ROI)	**9.2:1**	**2.8:1**

Staff costs (ANP and admin) are presented as full attribution for transparency. In practice, these staff also delivered other respiratory services, so true marginal costs are lower.

#### 2.4.2 Qualitative data collection & analysis

Semi-structured focus groups (*n* = 2 groups, 8 and 10 participants each) were conducted before service initiation. Surveys were conducted at each episode throughout the 20 months. Topics explored experiences of using the technology, feelings of safety/autonomy, interactions with the MDT, and the impact on daily life. Additionally, open-ended questions within the end-of-episode Virtual Ward Satisfaction Surveys provided supplemental text data.

Analysis was conducted using NVivo 12 software which coded the data into themes and sub-themes, quantified the sentiment, and provided a narrative summary (Table 4). Discussion among the multidisciplinary research team (clinicians, digital health technology providers and health service researchers) offered opportunity to challenge interpretations and ensure analytic credibility and self-awareness.

**Table 4:** Patient sentiment analysis from Virtual Ward Satisfaction Surveys (*n* = 159 episodes), showing proportions of positive and negative sentiment with representative patient quotes and identified key drivers/barriers..

Positive sentiment (55%)	Negative sentiment (20%)
"Excellent piece of kit"	"Hard to use because of vision"
"Very easy to use"	"BP wouldn't work"
"Great support"	"Fed up doing it"
Key Driver: The Watch & Nurse Contact	Key Barrier: Device Complexity & Carer Dependency

Quantitative and qualitative findings were integrated during the interpretation phase, to build a cohesive explanation. We used a weaving approach in the reporting, using qualitative narratives to explain, contextualise, and sometimes challenge the quantitative results. For instance, quantitative improvements in Borg scores were given meaning by qualitative narratives about the psychological difference between experiencing breathlessness in a familiar home environment vs. a frightening hospital ward (25). Discrepancies between high satisfaction scores and reports of “hidden labour” were explored as a key tension (26).

### 2.5 Patient and public involvement (PPI) and iterative co-design

PPI was a core, dynamic mechanism of the study, directly influencing the “embedding and adaptation” domain of the NASSS framework (18, 27). Initial PPI workshops with a local COPD support group informed the VCP's protocol, the choice of patient-facing language, and the design of training materials (22). Crucially, an iterative, responsive co-design process continued throughout implementation. A formalised “User Feedback Loop” was established: patient-reported issues—logged via the helpdesk, in surveys, or mentioned in clinical interactions—were collated and reviewed fortnightly by the project steering group.

Hardware issues were promptly addressed; for instance, following persistent reports of connectivity failures, a batch of thermometers with unreliable Bluetooth syncing was identified and replaced. Concurrently, the clinical team adapted the educational materials, developing and deploying simplified “how-to” video guides hosted directly on the tablet to address recurring patient queries. Flexibility was also built into the protocol itself; a manual data entry override was introduced to the tablet application, allowing patients to record observations during periods of connectivity failure and thereby reducing frustration. Finally, the digital infrastructure was calibrated to the patient cohort: the alert algorithms were fine-tuned to filter out transient, non-clinically significant physiological fluctuations, a change which served to minimise “alert fatigue” for both the patients and the multidisciplinary team.

This adaptive, learning-health-system approach was critical for improving usability and acceptability, demonstrating that the pathway itself was not a static intervention but a socio-technical system evolving in response to user experience.

## 3 Results

### 3.1 Participant characteristics and the digital inclusion profile

The enrolled cohort (*n* = 85) had a mean age of 68 years (SD ±9), with 55% male and 45% female. All participants (100%) owned and reported basic proficiency with a smartphone. This is a critical demographic finding, as it contrasts with national figures on digital exclusion (14). Over the 20-month active monitoring period, a total of 159 discrete exacerbation episodes were managed via the VCP. The cohort's digital readiness, while ensuring feasibility, flags a significant generalisability limitation and a central theme for discussion: the equity of access.

### 3.2 Quantitative outcomes: efficacy and utilisation

The virtual care platform demonstrated substantial impact on healthcare utilisation and patient-reported outcomes. Of the 159 exacerbation episodes managed on the platform, 155 (97.5%) were successfully completed without requiring hospital admission. Only four episodes (2.5%) required escalation to hospital care, triggered by clinical change—specifically new-onset atrial fibrillation detected remotely (*n* = 2), social circumstances (*n* = 1), and patient preference due to severe anxiety (*n* = 1). For those managed entirely at home, the mean length of stay on the platform—from episode initiation to discharge back to usual care—was 5.15 days (95% CI: 4.8–6.6). This represents a 51.5% reduction in observed length of stay compared to the pre-intervention regional baseline of 11.8 days for HIPE-classified “major severity” COPD admissions (*p* < 0.001) (Note: *p*-value derived from comparison of mean LOS between the VCP cohort and historical HIPE baseline; no concurrent control group was used) (4). It is important to clarify that this is not a direct patient-level comparison but rather a benchmarking against historical regional data for similarly comorbid populations. The reduction reflects the combined effect of the remote monitoring pathway, daily review by a specialist multidisciplinary respiratory team, and hospital-avoidance strategies—elements not routinely present in usual inpatient care, where non-respiratory teams often manage COPD admissions. This shorter duration likely reflects both the avoidance of hospital-associated delays, such as waiting for test results or discharge planning, and the efficacy of early, home-based intervention delivered by a specialist respiratory multidisciplinary team. The avoidance of hospital admissions translated into an estimated 1,004 inpatient bed days saved, based on the comparator length of stay. This estimate assumes that exacerbations meeting protocol-defined escalation criteria (failed rescue script, SpO₂ drop >4%, purulent sputum with positive culture, or MDT-determined deterioration) would have resulted in hospital admission under standard care. We acknowledge that not all AECOPDs in high-risk patients lead to admission; some are managed in primary care. Individual-level pre-post admission rates for each participant were not analysed, and therefore the bed-day estimate represents a potential upper bound rather than a definitive counterfactual.

Regional data from the National Clinical Programme for Respiratory Ireland provide supporting population-level signals. In the first year of the COPD Virtual Care Pathway's operation (2024), total bed days used for COPD admissions in the GUH catchment area fell by 570 compared with the preceding year (from 3,977 bed days in 2023 to 3,407 bed days in 2024). Additionally, the 90-day readmission rate for COPD in the region resulted in 17%, below the national average of 25% (35). It is important to note that these regional improvements may not be attributed solely to the Virtual Care Pathway; they likely reflect a combination of the VCP, the concurrent development of regional integrated care hubs, and other service enhancements. Nevertheless, the directional consistency between the episode-level bed-day estimate and the population-level reduction in bed day utilisation supports the feasibility of admission avoidance.

Applying the national Diagnosis-Related Group cost of €6,411 for a COPD admission without major complications, this equates to approximately €949,000 in direct hospital cost avoidance (15, 16). Against this, the estimated total service delivery cost over the 20-month pilot was €289,175 (comprising €106,800 in non-pay technology costs and €182,375 in pro-rated staffing costs). The resulting net cost avoidance was therefore approximately €659,825, representing a return on investment of 3.3:1 when staff costs are included, or 8.9:1 when considering only non-pay technology investment.

On a per-episode basis, the average cost of delivering the Virtual Care Pathway was €1,091 per exacerbation managed, compared with an estimated hospital admission cost of €6,411. Even when staff costs are fully attributed, the virtual pathway achieved substantial net savings. However, several caveats apply. First, these figures assume that exacerbations meeting escalation criteria would have resulted in admission—a conservative assumption discussed above. Second, the analysis does not include primary care costs or patient out-of-pocket expenses. Third, staff costs are based on whole-time equivalents assigned to the service and may overestimate marginal costs as staff have other clinical duties. A full incremental cost-effectiveness analysis is planned.

Patient-reported outcomes revealed statistically significant and clinically meaningful improvements over the course of each managed episode. To evaluate these changes, a paired *t*-test was applied, which compares pre- and post-treatment means within the same individuals to determine if the observed differences are statistically significant. Dyspnoea, as measured by the Borg scale, decreased by a mean of 3.2 points (from 6.1 to 2.9, paired *t*-test *p* = 1.028 × 10⁻⁹), exceeding the established minimal clinically important difference of 1.0 for this instrument (23). Similarly, COPD Assessment Test scores improved by a mean of 6.7 points (from 24.8 to 18.1, paired *t*-test *p* = 2.348 × 10⁻⁸), more than triple the minimal clinically important difference of 2.0 (24).These findings indicate that remote management not only kept patients out of hospital but also actively improved their acute symptom burden.

### 3.3 Qualitative findings: lived experience and mechanisms

Thematic analysis of focus group and survey data yielded three core, interrelated themes that illuminate the underlying mechanisms shaping patient engagement, experience, and the unintended consequences of “home hospital.” Overall sentiment towards the virtual care platform was predominantly positive, with 55% of survey comments expressing satisfaction using descriptors such as “excellent” and “brilliant.” However, beneath this favourable reception lay a more complex picture, revealing sub-themes.

Traumatic hospital experiences were a primary driver of acceptance and motivation. For the majority, the decision to engage with the virtual care platform was strongly motivated by negative, occasionally traumatic, past experiences of hospitalisation. Participants described hospitals not as places of rest and improvement, but as sites of “fear”, “loss of control”, and “loss of dignity”. This built in hesitation to hospital lowered psychological barriers to adopting unfamiliar technology (28). Fear of nosocomial infection was a dominant, almost universal concern. “Every time I go in with my chest, I catch something else”. This fear made the infection-control aspect of home care attractive. The depersonalising nature of hospitalisation was also a strong theme; “You use a commode on the corridor”. The virtual care platform was seen as a way to retain dignity and identity. Furthermore, the hospital environment itself was described as a barrier to recovery; “The noise, the lights you don't get a wink of sleep”. The comfort and quiet of home were framed as therapeutic in themselves.

The Autonomy-Safety Tension: The RPM was a “Digital Safety Net” but also a source of new anxieties. Participants expressed a strong, yet paradoxical, duality in their feelings about the virtual care platform. They cherished the autonomy and “normality” of staying at home—“being able to sit in my own kitchen, look out at my own garden”. However, this cherished autonomy was closely linked with underlying anxiety about the potential for rapid deterioration. The remote monitoring technology directly mediated this tension (26). The technology was regularly described in as a “safety net”, or “having a personal nurse”. The knowledge that physiological data was being watched by experts provided reassurance that enabled them to feel safe at home. This reassurance was closely tied to the human element of the service; participants regularly differentiated between the technology and the clinical team, with satisfaction often attributed to the “reassurance” and “comfort” provided by nurses, suggesting that the human element may compensate for technological shortcomings. However, this dependency created a new, technology-focused form of anxiety. The “safety net” was perceived as fragile. Anxiety spiked when devices failed to sync—reflected in survey comments expressing uncertainty about whether observations were received. The fear of technical failure became linked with fear of clinical abandonment (29).

The Hidden Labour and Burden of “Home Hospital” was themed**.** An unanticipated finding was the workload transferred to patients and their family carers. Participants described this as the “hidden job” or “the admin” (26). Technical barriers remained, including difficulties with peripheral devices such as thermometers, and confusion regarding data transmission. Patients became responsible for managing a mini-IT ecosystem: troubleshooting Bluetooth connections, ensuring charging of devices, and resolving syncing errors. The intervention revealed a reliance on what we term “digital proxies” —family members, particularly grandchildren and daughters or sons who were essential in facilitating the technology on occasion. Survey data indicated that tasks were performed by daughters, grandsons, or carers, with one participant noting their “grandson was using the app not me”. This reliance, while effective, introduced significant carer burden, adding to the existing unpaid care work of family members. Participants estimated spending 15–20 min daily on these tasks during an exacerbation, a burden notably higher and stressful for those who described themselves as “not tech-savvy”. This finding reveals that device ownership and access do not equate to effortless digital health literacy, and that the successful operation of home hospital models often depends on invisible, unpaid labour (7).

### 3.4 Iterative refinements: evidence of a learning system

Throughout the implementation phase, a responsive co-design approach ensured that the virtual care pathway evolved in direct response to user feedback and clinical experience, yielding several tangible improvements that enhanced overall feasibility (27). Hardware optimisation was undertaken following user reports of faults with the original thermometers during the initial months; these devices were subsequently replaced, after which no further issues with temperature recording were reported. Concurrently, the tablet application was enhanced through the addition of a “manual entry” function, enabling patients to record and transmit observations during temporary connectivity failures, a modification that substantially reduced user frustration. Recognising the need for accessible guidance, the clinical team developed a series of short, targeted video tutorials addressing the most frequently recurring patient queries, for instance Airway clearance techniques, which improved user self-management. Finally, the clinical protocol itself was calibrated in response to multidisciplinary team feedback: alert thresholds were adjusted to minimise non-actionable alarms, leading to a marked reduction in alerts that did not require clinical intervention. This refinement served a dual purpose, decreasing anxiety for patients while simultaneously reducing cognitive workload for the clinical team. Collectively, these iterative improvements underscore the value of embedding responsiveness within digital health implementation, ensuring the technology remained aligned with the practical realities of home-based care.

## 4 Discussion

This feasibility study demonstrates that a hospital-initiated VCP for COPD exacerbations can be successfully operationalised in an Irish setting, yielding impressive quantitative results: a reduced LOS, a 97% reduced presentation to emergency department and statistically and clinically significant improvements in patient-reported dyspnoea and symptom burden (9, 11). These findings align robustly with international evidence on RPM for COPD and offer a tangible, proof-of-concept model for operationalising Sláintecare's “right care, right place, right time” ambition (8). However, the critical realist lens and our rich mixed-methods data compel us to look beyond these surface-level successes and interrogate the deeper, systemic mechanisms at play that will ultimately determine whether this innovation reduces or reproduces inequity and whether it can be sustainably scaled. The discussion therefore centres on two paramount, interrelated tensions at the heart of Ireland's digital health future, namely the Equity Paradox and the threat of an “Inverse Digital Care Law”, together with the paradox of a hospital-led 'shift left' and the sustainability challenge it presents.

### 4.1 The equity paradox and the threat of an “Inverse Digital Care Law”

Our study reveals a profound equity paradox that stands as the most critical finding for policymakers. While it is striking that every participant reported owning a smartphone, this was not a function of our recruitment strategy. All patients referred to the service were offered enrolment and, crucially, were provided with a tablet as part of the remote monitoring kit—a device with built-in 5G connectivity operating independently of personal internet access. Smartphone ownership therefore neither dictated nor influenced who was onboarded; we inquired about prior device experience solely to gauge whether participants might require additional support from clinicians.

However, the fact that every participant nonetheless reported owning a smartphone exposes the central premise of the equity paradox: our findings are inherently limited to a fully connected population. In this context, universal smartphone access is not a methodological artefact of *who was recruited*, but rather a stark illustration of *who is currently reached by*—and who remains excluded from—digital health research. The absence of digitally excluded voices from our sample is, in itself, the finding.

National data underscores this silence: approximately 37% of those aged 65+ in Ireland are digitally excluded, with rates soaring among those with lower educational attainment, lower income, and in rural areas with poor broadband (14). Significantly, COPD prevalence is itself socially patterned, often higher in these very disadvantaged communities due to historical smoking patterns, occupational exposures, and poorer living conditions (30). Consequently, the population at the greatest clinical risk for frequent, costly exacerbations is also the population most likely to be excluded from a purely digital solution.

We posit, drawing on substantial qualitative evidence regarding “hidden labour” and digital anxiety, that without deliberate, proactive, and funded intervention, Ireland risks cementing an “Inverse Digital Care Law” (7). In this scenario, digitally enabled, convenient, and effective care becomes most accessible to those who are already more resourced, health-literate, and technologically confident. Meanwhile, those with the greatest multi-morbidity, socio-economic complexity, and highest needs remain trapped within a struggling, reactive acute hospital system. This concern is powerfully echoed in contemporary scholarship (31). Andersson et al. (7) argue that digital health innovations can inadvertently widen health inequalities if they are designed for and evaluated with “highly selected, motivated, and digitally literate patients”—the unintentional cohort of this pilot. Our own co-design process, while beneficial for engaged users, may have inadvertently tailored the model away from the needs of non-users, creating a feedback loop that reinforces exclusion.

To prevent this inequitable future, scaling must be preceded by the proactive, mandatory design of hybrid (digital-analogue) care pathways, designed not as fall backs but as core, equitable components. For the digitally included, this would entail continuing and refining the technology-supported pathways successfully piloted in this study. For those who own devices but possess lower digital literacy, technology-facilitated pathways must be established, integrating intensive, ongoing support from community link workers or “digital navigators” to bridge the confidence and skills gap. Most importantly, for the digitally excluded, fully integrated low-tech alternatives—such as structured daily telephone support delivered by a community nurse using the same clinical protocol as the digital platform—must be embedded within the pathway to ensure these patients receive care with the same timeliness and clinical rigour as their digitally connected counterparts. Encouragingly, as the pilot progressed, clinicians began adapting their workflows organically to overcome digital literacy challenges encountered in real time, this included structured daily telephone support where required or identified, thereby reducing exclusion from the service offer from the outset.

As Meyer et al. (32) argue in *Frontiers in Digital Health*, “equity must be a primary design feature, not an afterthought”. This requires dedicated funding lines, mandatory inclusion of underserved groups in co-design, and the of development of performance metrics that explicitly track who is reached and how they fare, across digital literacy and socio-economic groups.

### 4.2 The paradox of hospital-led “Shift Left” and the sustainability challenge

The second major tension lies in the model's alignment with the fundamental goals of system reform. While the VCP clinically delivers care “in the right place” (the home), its governance, funding, and clinical leadership in this pilot present a complex picture that reflects the fragmented nature of current service arrangements. The advanced nurse practitioner occupies a hospital-funded post with a service level agreement to cover community services on a 50:50 basis, while the consultant post is community-funded with a corresponding service level agreement to cover acute services. The remaining team members, the COPD outreach staff, hold full hospital-funded posts yet are clinically governed by the integrated care consultant. This creates a strategic paradox: a reform aimed at de-centering the hospital is, in this instance, being delivered through a patchwork of funding streams that blur the boundaries between acute and community sectors, raising critical questions about long-term sustainability, scale, and incentives that reward cost-shifting rather than integrated care.

For VCPs to become mainstream and truly integrated, the governance, funding, and clinical leadership must “shift left” to the planned Integrated Care Programmes and the foundational Community Healthcare Networks. Encouragingly, the emerging roadmap for integrated care services in chronic disease prevention and management, aligned with the restructuring of health regions, proposes central funding for service provision. While this reform remains in its infancy, it represents a welcome departure from the artificial divide of calculating savings by sector; the patient cohort is shared, and they deserve optimal care regardless of whether it is delivered in acute or community settings. The sustainable funding model cannot therefore be one of simple cost avoidance for hospitals, which creates a budgetary silo problem. Instead, it requires a bundled payment for the holistic management of a defined COPD population. This pooled budget, held by a Regional Health Care Organisation, would incentivise primary and community care providers to invest in and operate remote monitoring services, as they would directly benefit from keeping people well at home.

The economic analysis must therefore evolve from simple cost-avoidance to a full societal cost-consequence analysis over a multi-annual timeframe (15, 16). This analysis must incorporate: the recurrent costs of software licenses, hardware refreshes and replacements, 24/7 clinical monitoring capacity, community workforce development, and the costs of parallel analogue pathways for the digitally excluded. Boland et al. (16) caution that the business case for digital health often fails in systems like Ireland's precisely because savings in one budget (hospitals) do not translate into investable capital for another (community care or digital infrastructure). Breaking this cycle is essential for scale.

### 4.3 Limitations

This study has several important limitations that qualify the interpretation of its findings and point to essential avenues for future research (33). First, the absence of a concurrently randomised control group limits our capacity to make definitive claims about the programme's effectiveness relative to standard care. While historical HIPE data provide a pragmatic benchmark for similar-severity admissions, the reported 51.5% reduction in length of stay should be interpreted as a real-world effectiveness marker against regional averages rather than a direct comparative efficacy estimate. The observed difference is influenced by differences in care delivery models (specialist respiratory team vs. general medical ward care) and episode definitions, in addition to any true treatment effect. A definitive randomised controlled trial is warranted to establish efficacy with greater methodological rigour. Second, and most significantly, the issue of selection bias constrains the generalisability of our findings. The technological capacity required for participation, whether directly or with support, means that our promising results are not readily transferable to the wider COPD population, particularly those experiencing digital exclusion. This limitation is not merely methodological but substantive, reinforcing the equity paradox discussed throughout this paper. Third, the study's duration and scope capture only short-to-medium term outcomes; long-term impacts on disease progression, mortality, and patient and carer quality of life remain unknown. Furthermore, the analysis presented here reports only a subset of patient-reported outcome measures; full analysis of health confidence, self-efficacy, and detailed usability metrics is ongoing. Finally, as a single-site pilot conducted within one regional hub, the findings may not fully reflect the operational challenges encountered in other Irish regions, where community health infrastructures and digital connectivity vary considerably (13).

Moreover, the estimated 1,004 bed days saved rests on an assumption that exacerbations meeting physiological escalation criteria would have resulted in hospital admission under standard care. We did not capture individual participants' AECOPD frequency in primary care nor the proportion of prior exacerbations that led to hospitalisation in the year before enrolment. Some exacerbations managed on the RPM platform might have been managed with rescue medication in primary care. Consequently, the bed-day and cost-avoidance figures should be interpreted as potential upper-bound estimates rather than definitive savings. Future studies should include individual-level pre-post admission rate analysis and primary care linkage to refine this assumption.

Finally, our cost estimates are based on service-level budgeting rather than marginal cost analysis. The reported €1,068 per-episode cost includes pro-rated staff salaries that may not fully reflect the opportunity cost of redeploying existing clinical staff to the virtual pathway. Conversely, the cost-avoidance figure of €6,411 per admission uses a national average Diagnosis-Related Group rate, which may not capture the full marginal cost of an avoided admission (e.g., fixed costs such as hospital infrastructure that remain regardless of occupancy). Additionally, we did not quantify costs incurred by primary care, community nursing, or patients’ own expenses (e.g., heating, electricity, or informal care time). Future economic evaluations should adopt a societal perspective and include sensitivity analyses around these parameters.

Collectively, these limitations underscore the need for multisite, adequately powered trials that are deliberately designed to include and examine outcomes for digitally excluded populations.

## 5 Conclusion

This pilot study confirms the technical and clinical feasibility of a nurse-led Virtual Care Pathway for COPD exacerbations in Ireland and demonstrates its potential to produce superior utilisation and patient-reported outcomes for a digitally included, motivated patient group (12).

However, it also set out to investigate not simply whether a virtual care pathway for COPD exacerbations could work in an Irish context, but how, for whom, and under what conditions it might succeed or fail within a fragmented health system navigating a historic reform agenda. By adopting a critical realist lens and applying the NASSS framework, we have moved beyond a narrow evaluation of clinical efficacy to expose the deeper socio-technical mechanisms that will ultimately determine whether Ireland's digital health transformation fulfils its promise or deepens inequalities.

The quantitative findings affirm the clinical potential of a virtual care pathway for COPD. A 97% success rate for patients managed within the Virtual Care Pathway, a 51.5% shorter observed length of stay relative to regional historical baselines for similarly comorbid COPD admissions, and clinically meaningful improvements in both dyspnoea and COPD Assessment Test scores demonstrate that, for a selected cohort, hospital-initiated remote monitoring can safely and effectively manage acute exacerbations in the home. These outcomes align with the strongest international evidence and provide a tangible proof-of-concept for operationalising Sláintecare's vision of delivering “the right care, in the right place, at the right time” (8, 9, 34). The estimated €949,000 in direct hospital cost avoidance further underscores the potential economic case for scaled adoption, offering a glimpse of what a reoriented health system might achieve.

The narrative of success suggested by satisfaction rates and metaphors of the “safety net” is enriched and complicated by our qualitative data. It shows that the virtual care pathway functions as a socio-technical system, where care provision is within a network of people and technology. This dynamic, while effective, implies a transfer of certain responsibilities to the caregivers, including the “hidden job” of ensuring connectivity and quantifying subjective experiences. The key role of families acting as “digital proxies”—grandchildren and daughters or sons—brings the generational nature of this care work into focus. Acknowledging these dimensions is not to diminish the service achievements, but to fully understand the ecosystem it creates and to proactively design support for the families who are potentially pivotal to its success (26, 36)”.

Most critically, this study surfaces a fundamental equity paradox that must confront Irish policymakers and health service leaders. Our cohort, universally connected and digitally capable, represents precisely the population least at risk of exclusion. Yet COPD, like many chronic conditions, is socially patterned; its highest burdens fall on older, poorer, and more marginalised communities—the very groups most likely to be digitally excluded (14, 30). The absence of these voices from our sample is not a methodological limitation to be ignored. Without deliberate, funded, and mandated action, Ireland risks enacting an “Inverse Digital Care Law” in which those with the greatest clinical need remain within a struggling, reactive hospital system while the digitally enabled receive proactive care at home (31, 36).

The path forward requires a fundamental shift in how virtual care is designed, governed, and funded, moving beyond the logic of pilots to a sustained, equity-focused programme of implementation. At the heart of this transformation must be a commitment to equity as a design imperative. This means that hybrid pathways cannot be treated as fallback options for those excluded from technology; they must be core, equivalently resourced components of any national virtual care service. For the digitally excluded, this means embedding structured telephone support—delivered by community nursing teams and following the same clinical protocols as the digital pathway—as a parallel offer. For those with low digital confidence, it requires investing in funded “digital navigator” roles, whether community link workers, family carers, or trained volunteers, to provide the sustained, relationship-based support that enables genuine participation.

Resolving these equity challenges is inseparable from resolving the governance and funding paradox exposed in this pilot. Sustainable virtual care cannot be achieved through a hospital-led model of “shifting left”; it requires that clinical leadership, governance, and crucially, budgets reside within the community and integrated care structures that are the intended future backbone of the Irish health system.

Achieving this alignment also demands that we embed the adaptive, learning system principles that enabled iterative refinement during this pilot. Digital health technologies are not static interventions to be installed and left alone; they are evolving networks of people, devices, protocols, and relationships. Their successful embedding at scale requires ongoing adaptation, responsive governance, and robust mechanisms for capturing and acting upon user experience in real time. This capacity for continuous learning—whether through hardware replacement, protocol calibration, or rapid feedback loops—must be resourced not as an afterthought but as a core, dedicated component of any implementation budget.

The study's limitations, including its single-site design and selected cohort, point clearly to the next research agenda. Multisite, adequately powered trials are needed. There needs to be stratified sampling to ensure meaningful representation from those with limited connectivity, low digital literacy, and high social complexity. The outcomes measured must extend beyond clinical metrics to encompass carer burden, digital confidence and equity impacts. Long-term follow-up is essential to understanding impacts on disease progression, mortality, and the sustainability of behaviour change, while full economic evaluations must capture costs across all sectors, including the hidden costs of unpaid care work and the parallel analogue pathways required for equitable access.

For Irish policymakers, the implications of this work are both necessitated and actionable. The digital health infrastructure piloted here is not a possibility but a present reality to being scaled across the country. The defining question is therefore no longer whether virtual care for COPD can work, but for whom it works, at what true cost, and with what consequences for equity. As the Health Service Executive advances its national digital health strategy and the Regional Health Areas begin to assume operational responsibility, the findings of this study offer both a roadmap and an insight. The roadmap demonstrates that virtual care, when clinically led, responsively designed, and adequately resourced, can deliver outcomes that align with international best practice and the core ambitions of national reform. The insight is also clear, digital transformation will not bridge the health divide but become a new inequality if not handled correctly.

## Data Availability

The original contributions presented in the study are included in the article/Supplementary Material, further inquiries can be directed to the corresponding author.
